# What Impact Does Participation in a Communication Skills Training Program Have on Health Professionals’ Communication Behaviors: Findings from a Qualitative Study

**DOI:** 10.1007/s13187-023-02305-9

**Published:** 2023-05-08

**Authors:** V. White, M. Chiswell, E. Webber, P. Martin, A. Piper

**Affiliations:** 1 Deakin University, 1 Gheringhap St, Geelong, Victoria Australia; 2 Cancer Council Victoria, 615 St Kilda Rd, Melbourne, Victoria Australia

**Keywords:** Communication skills training, Health professionals, Qualitative interviews, Impact, Barriers

## Abstract

Communication skills training can enhance health professionals’ knowledge and repertoire of effective communication practices. This paper describes the conceptual model underlying a 3-day retreat communication skills training program, methods used for training, and participant perception of outcomes from the training using qualitative interviews. Repeated qualitative telephone interviews (approximately 6 months apart) with participants of a 3-day Clinical Consultation Skills Retreat. Fourteen participants (70% response, 57% doctors) took part at Time 1, with 12 participating at Time 2. Semi-structured interviews were recorded and transcribed, and directional content analysis was conducted to assess themes in areas of key learnings, implementation of skills, and barriers. The training was received very positively with participants valuing the small group learning, role play, and facilitator skills. Key learnings were grouped into two themes: (i) tips and strategies to use in clinical practice and (ii) communication frameworks/methods, with the second theme reflecting an awareness of different communication styles. Most participants had tried to implement their new skills, with implementation reported as a more deliberate activity at T1 than at T2. Those implementing the new skills noted more open conversations with patients. Practical barriers of lack of time and expectations of others were mentioned more often at T2. A 3-day retreat-based communication training program was positively received and had a positive impact on the use of new communication skills. While further work is needed to determine whether effects of training are evidenced in objective clinical behaviors, the positive longer-term benefits found suggest this work would be worthwhile.

**Supplementary Information:**

The online version contains supplementary material available at 10.1007/s13187-023-02305-9.

## Introduction

Effective communication between health professionals and patients and their family members/carers is recognized as an essential component of quality health care [1]. To this end, in 2017, the American Society of Clinical Oncology released consensus guidelines for patient-clinician communication highlighting the role patient-centered communication has in improving outcomes for patients [2]. In Australia, effective patient-health professional communication is recognized as a core component of quality cancer care, with federal and state government-endorsed optimal care pathways (OCP) recognizing health care systems have an obligation to meet patient communication needs [3].

Health professionals often need additional support to assist them in their communications with patients, especially in the delivery of bad or difficult news [4]. Communication skills training has been advocated as a strategy to enhance health professionals’ knowledge and repertoire regarding effective communication practices [2, 5], and the latest version of Australia’s OCPs recognizes the potential of training to enhance clinicians’ communication skills. While many programs have been developed, components are generally similar and include defining essential components for effective communication (e.g., use of open questions, conveying empathy, comfort with silence), learning through observation, role-play, and practice and structured feedback and self-reflection [6, 7]. Small group learning and skilled facilitators to model the desired communication skills and facilitate experiential learning exercises are also recommended [6, 7].

**Fig. 1:**
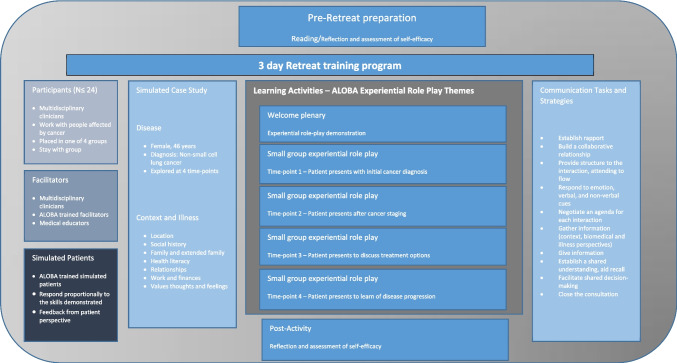
Description of the content and format of the 3-day communication training retreat

This paper reports the experiences of oncology healthcare professionals’ participation in a 3-day communication training program run as a retreat (referred to as the retreat). The retreat provides the opportunity for participants to apply core communication skills across the care trajectory—from diagnosis to end-of-life. Using small group learning principals that involved 4–6 participants working with one facilitator and a learner-centered approach, participants identify individual communication challenges with experiential role-play tailored to reflect their needs, interests, and health professional backgrounds. Simulated case studies containing both disease and illness elements are used to ensure participants practice patient-centered clinical interviewing [2, 8]. The simulated case (played by paid actors) are rich with contextual factors (e.g., responsibilities, social support, and access to care) to ensure an understanding of the patient’s context including an exploration of values and goals and how these factors influence care planning and decision-making [9]. The case study is visited at various time points in clinical care, with common themes arising at each time point such as responding to emotion or facilitating shared decision-making, identified areas for exploration. The Calgary-Cambridge Agenda Led Outcome-Based Analysis (ALOBA) model is used to structure and facilitate communication skills training [10] (see Fig. 1 for structure and content of the retreat). Facilitators were senior clinician-academics with between 15 and 25 years of experience in delivering health communication training to a diverse range of health professionals and disciplines.

This paper presents findings from qualitative interviews to assess the impact of participation in the retreat on communication behaviors in the clinic in the short and longer term and to understand barriers participants identify to implementing their skills in the clinic.

## Method

### Design

Longitudinal, qualitative telephone interviews with participants from a 2019 Clinical Consultation Skills Retreat (the retreat). Qualitative method from a critical realist position was employed to allow a deeper understanding of participants’ learning experiences and implementation of skills [11].

### Retreat Training

The retreat ran from Monday to Wednesday at a regional conference center, with participants staying onsite.

### Recruitment

At the end of the retreat, participants were provided with information about the study including participating in two telephone interviews. Interested participants provided written consent to be contacted about the interviews.

### Interviews

Semi-structured interviews included open-ended questions assessing experiences and outcomes from the retreat. Open-ended questions in Interview 1 (T1, approximately 4–6 weeks post-training) assessed impressions of the training program, including facilitators, use of actors for the role plays, and small group learning. Similar open-ended questions were used in Interview 2 (T2, 6 months after T1) to assess key learnings, implementation of learnings, outcomes, and barriers. All interviews were conducted by the same female behavioral researcher (Author 1) with a PhD and over 20 years of experience working in cancer control and support. Interview 1 lasted on average 31 (range: 21–41) min, and Interview 2 had an average of 21 (range: 12–31) min (see Supplementary Table 1 for relevant T1 and T2 interview questions).

### Procedure

Potential participants were contacted by email from Author 1 (interviewer), provided with the study’s plain language statement, and invited to take part. Interview times were arranged after study consent obtained.

Consenting participants were contacted at the agreed time, and after obtaining verbal consent for audio recording, the interview commenced. At interview end, participants reconfirmed consent to take part in the second interview. Procedures for the second interview followed those for Interview 1, with the interviewer contacting participants by email and after obtaining consent, arranging an interview time. Interviews were conducted over the telephone and audio recorded with participant consent. The study had ethical approval from the organization ethics committee (IER: 1914). Reporting follows the COREQ guidelines.

### Analysis

Audio recordings were transcribed and inductive, reflexive thematic analysis undertaken [11] with NVivo™ used to manage data and coding. Interviews were considered both cross-sectionally (i.e., Interview 1 or 2) and longitudinally for each participant. Three predetermined topic areas were established reflecting the interview schedule and study aims: “Key learnings,” “Implementation of learnings, outcomes and barriers,” and “Perceptions of Format” (Interview 1 only). After data familiarization, a coding scheme was developed (Author 1) which was reviewed, tested, and discussed with all team members. After the finalization of the coding scheme, the data were coded, and themes and subthemes were developed with discussion and checking at a team level. The saturation of major themes identified for each topic area was reached.

## Results

### Participants

Twenty people participated in the training program, with 14 taking part at T1 (response rate 70%, 11 (8 (79%) female)) and 12 participating (10 (83%) female) at T2. Eight participants were medical doctors from a range of specialities including anesthesia (*n*=2), geriatrics (*n*=1) palliative care (*n*=2), pain management (*n*=1), radiation oncology (*n*=1), and intensive care (*n*=1). Other participants included nurses (*n*=4), social work (*n*=1), and hospital management (*n*=2). Retreat attendance for three participants was funded by their employer; three (two nurses, 1 doctor) received grants to attend, with the remaining participants using Continuing Medical Education funding 1 to fund participation.

#### Topic Area 1: Key Learnings

Key learnings were grouped into two main themes: (i) skills/strategies to employ in clinical practice and (ii) communication frameworks/methodology. Table 1 presents the key learnings from the two interviews. Theme 1 reflected participants’ focus on the communication skills (e.g., value of silence/allowing pauses (*n*=5)) learned through the retreat. There was some change in the skills mentioned over time: “pausing” was common at both interviews, while “listening,” “chunking and checking,” and the use of “I wish” statements were mainly mentioned at T2. The second theme, “communication frameworks/methodology,” reflects participants growing awareness of communication methods and frameworks, their own communication style, and how they respond to different conversations. Exemplar quotes are as follows:

**Table 1:** Key learnings from the retreat mentioned at Interview 1 and Interview 2 for participants (multiple responses from participants allowed)

Interview 1	*N*	Interview 2	*N*
Skills/strategies to use within conversations			
Value of silence/allowing pauses	5	Taking time--pausing	4
Reflecting/responding to emotions,	2	Seeing things from the patient’s point of view/ask don’t tell/reflecting patient emotions	3
Probing why a question was asked, rather than just answering it	2	Importance of listening	2
Listening	2	Use of “I wish” statements	2
Asking open questions	1	Chunking information	2
Sitting with discomfort	1	Importance of open questions	1
Importance of summaries	1	Being clear about what happens next/what the next steps are	1
		Attending to cues in the conversation	1
		Sitting with ambivalence in the conversation	1
		Signposting what is coming up in conversation	1
		Being clear: not being afraid to use the actual word, not camouflaging what you’re saying	1
		Awareness of non-verbal communication	1
Enhanced self-awareness			
Awareness of own communication style	2	Awareness of own communication style	1
How sentences are structured		Awareness of speaking too fast, the need to slow down speech	2
Awareness that agenda of patient and health professional may differ, need to align agendas	1	Ability to reflect and acknowledge my response and where I am when engaging with someone	1
What health professionals say can impact patients	1		
Small changes can have a big impact	1		
Reflecting on own emotions/ responses			
Methodology			
Frameworks for communication	4	Methodology of communication skills	2
Learning from observing others	3		
Tools in your toolkit to pull out to try	2		
Preparation			
		To think through a conversation rather than just approach it ad hoc/preparation	4
		Setting up a space and room	1

*My key learnings were probably around how I might approach difficult conversations or how I might structure my sentences and the way I might ask a question or try and tease out more information from someone*. *(T1) (social worker)*

*The key learnings were the methodology of the communication skills, for example, different tools to use for communicating with patients. Something I find I do a lot more is chunking information to patients. I find that a very useful tool. (T2) (specialist doctor)*

#### Topic Area 2: ‘Implementation of learnings, outcomes and barriers’

##### Implementing Skills into Practice

Most participants had tried to implement the communication skills into practice. While this was seen as a more deliberate activity at T1, by T2, many skills were employed more regularly. At each interview, several participants discussed the implementation of communication skills in relation to them having greater awareness of their communication practices.

##### Communication Outcomes

Two themes were identified: “patient outcomes” and “communication with other professionals.” In the “patient outcomes” theme, participants discussed how the new skills had allowed more open and responsive conversations with patients, which allowed patients to raise issues that they thought would not be discussed otherwise. For those in palliative care, the new skills enabled the opening up of end-of-life care discussions. The second theme “communication with other professionals” reflected the utility of the communication skills in their broader professional life including conversations with staff and in developing and maintaining relationships with colleagues (see Table 2 for exemplar quotes).

**Table 2:** Exemplar quotes for the impact of implementing the communication skills in the themes of (i) patient impact and (ii) staff impact at Interview 1 (T1) and Interview 2 (T2)

	Exemplar quotes
Patient impact	
T1	I had one patient where I had met for the first time and he came out of the consultation and sort of said, “I have never revealed this particular issue to anyone before.” So he had been seen by multiple medical oncologists, radiation oncologists, psychologists, which he didn’t feel he kind of connected with. In that instance, it was a slightly surprising outcome, that he felt he was able to open up and discuss an issue particularly troubling him. (Doctor)
	I left a lot of spaces in the conversation for them to process. I also broke up the conversation into two separate events so that I gave them more time so that they weren’t overwhelmed, which is something I learned from the course, just about not just dumping and leaving. And the feedback from—and also choosing to use direct terms with them about death and dying and the feedback from them both was I was the first doctor who had ever mentioned the word dying with them. And they were both very relieved that they had both been thinking it and not talking to each other or talking to anyone else about it, so to have it out on the table they felt was a bit of a relief. (Doctor)
	I had a patient just recently, she’s pretty unwell and I was talking to her about her pain and things like that. And I just got a sense that there was more on her mind about what was going on and so instead of trying to ask more focused questions on the pain, I asked a more open question and gave her a bit of time to answer. And she was able to open up about her worries that she wasn’t going to make it to Christmas and that making it to Christmas is really important to her, but she’s worried that she’s not going to make it. And I felt like I wouldn’t have got that if I had just focused on her pain and management of her pain and not given her that chance to explore her feelings more. (Nurse/Allied Health)
	It was the first time I had spoken with [male carer] so I made sure I listened to everything he was saying in detail and I wrote a lot of notes and left a lot of pauses and slowed down what I was saying to him, so I could pick up on cues and ask those questions “tell me a bit more about that” or “you mentioned this can you explain that to me a bit more.” ….. At the end of the conversation, I explained my role to him and said that I am available for emotional support, ongoing support while his wife is receiving treatment. He said, “Yeah, actually you’ve been really helpful already today.” (Nurse/Allied Health)
T2	Today I saw a terminal patient. I guess my approach to it was to slow the consult down, not rushing or being this brand-new doctor dispensing advice. It was just to take some time to come in slowly, acknowledge everybody in the room, make sure they were comfortable with me and a med student being present. And then to explore what it meant to them to have their father, husband, dying and to watch them settle down and go, “Oh, this is a safe environment, we can talk.” (Doctor)
	I could see that we had to probably stop her treatment soon because she was getting too many toxicities from it and it was affecting her quality of life. Just utilizing those skills to be able to move her forward in her thinking, asking “What if she couldn’t go on with her treatment, because of the peripheral neuropathy?” It facilitated a conversation about there being other treatments, but we are coming to the end of the options, and is now a good time to think about, or talk about preparing for the event where you may be passing away soon or an advanced care planning, and we got into that conversation. (Nurse/Allied Health)
	With the comm skills course I spent time at the start of the consultation just letting him talk to me about what he was concerned about and went into some personal issues and let him run with it for a bit. I still achieved my objective of getting through the consenting process [for medical procedure], but it was actually, from a personal point of view, a far more rewarding consult because I think he walked out of the door a little bit more chirpy than he when he arrived. (Doctor)
Communication with other professionals	
T1	A team member who I needed to talk to about their performance and so preparing for that I used a lot of the work [from the training] and thinking about what I was going to say and how I was going to say it. So I found that very useful, when you can take that time to think about it and reflect on, then later, what you did. (Nurse/Allied Health)
	[talking to a staff member] I said “I get a sense that you are uneasy about something” and they said “yes” and I said “can you tell me more” and this whole thing opened and the staff member said at the end “I just feel much better for having got that off my chest”. (Doctor)
	One of the other things I probably gained that I hadn’t quite realized was these skills relate to not just your engagement with patients but with fellow staff. Just the value that it can have in having better, more meaningful conversations and communication with your peers and bosses (Nurse/Allied Health)
T2	We were going to have to sit down [with a team member] and have very difficult conversations [relating to ongoing work]. I was going to draw on the structures that I learned from the workshop. First to signpost to say “this is a difficult conversation, it’s probably going to be a bit difficult for both of us and this is what it’s about.” And then do my pause and ask them the question; what are your plans [relating to ongoing work] what are you thinking? (Nurse/Allied Health)
	Most skills are applicable across the board, so deep listening, been reading about deep listening. But also trying to pick up cues and all those things are applicable when you’re interacting with your fellow staff members. (Doctor)

##### Barriers

At T1, most participants thought they were able to implement the new learnings into clinical practice, although there was recognition of factors that could make this more difficult. A key barrier reported at T1 and T2 was the clinical role participants had, with this most evident in comments by intensive care specialists and anesthetists. Anesthetists indicated a lack of time due to multiple clinicians needing to see patients before surgery.

At T2 there was greater recognition of the practical barriers with more participants mentioning a lack of time due to the pace and demands of work, expectations of other health professionals, and patients as barriers. Some noted that the pace of work meant they could not plan conversations ahead of time and reverted to their old practices.

#### Topic 3: Perceptions of the Retreat’s Format

Comments on the retreat’s format were categorized into three main areas. Exemplar quotes for each area are shown in Supplementary Table 2.

##### Small Group Learning

This approach enabled the development of trust and allowed participants to feel “safe making mistakes” and “be more authentic.” Several felt they would not have been able to try new approaches if they were constantly changing groups. Most groups consisted of the same health profession, with one including a mix of doctors, nurses, and allied health professionals. While some in this group commented the mix reflected the multidisciplinary nature of oncology care others expressed some initial concerns. However all thought the facilitator’s skills ensured scenarios were challenging and relevant to all group members, and all were satisfied with the learnings achieved.

##### Simulated Patients

All participants thought the simulated patients were integral to the success of the training program as they enabled the experiential learning to reflect practice as much as possible and allowed skills learned to more easily transfer into clinical practice.

##### Facilitators

Participants noted facilitators were highly skilled communicators both clinically and as educators, and this assisted in the overall experience of the retreat.

## Discussion

Using qualitative interviews at two time points, this study examined the immediate and longer-term impact of participation in a 3-day retreat-style communication skills training program on the communication practices of oncology health professionals. Similar to others [12–15], the training program assessed had an immediate positive impact on health professionals’ awareness of their communication styles and use of new communication skills. Data from the second interview suggests that while, with time, the new skills became more internalized, there was also greater awareness of the barriers to using these skills, with the key barriers being the type of work professionals were involved with, patient and colleagues’ expectations, and the busyness of clinical practice. While further work is needed to determine whether the impact of the program found here is evidenced in objective clinical behaviors, the positive longer-term benefits reported by participants suggest this work would be worthwhile.

Careful consideration was given to the training’s format, the development of the simulated case study, the choice of facilitators, actors, and learning activities. Skills-based communication approaches aim to change communication behavior, requiring experiential practice to allow clinicians to practice skills, and to see how they can be integrated into practice. The ALOBA methodology for experiential role play provides a framework for analyzing simulated clinical encounters and giving feedback that maximizes learning and learner safety [16]. The retreat’s learner-centric approach and use of experiential learning with simulated patients were seen to be effective by all participants. The facilitators’ skills in running the small group learning were integral and frequently mentioned as a strength of the training.

The retreat was made available to any health professional working in oncology allowing health professionals from a range of specialty disciplines to attend. While the majority of attendees and study participants were medical doctors, nurses, allied health, and hospital administrators attended the retreat and were interviewed for this study. The number of participants at the retreat was capped to ensure groups of up to 6 participants worked with an assigned facilitator across the entire retreat. As most retreat participants were doctors, most groups contained only doctors. Only one group contained a mix of different health professional disciplines. Given the multidisciplinary nature of oncology care developing communication programs that enable different specialities to learn together may promote awareness of the different skills different health professions bring to the care of patients and the benefits of all being skilled communicators. The similarity of experiences and impact of the training across different health professionals found in our study suggests that the retreat’s learner-centric approach is effective regardless of the health professional background.

Findings from the two interviews suggested there was an integration of skills into regular practice with time. At T1, most participants spoke about including the new skills into clinical practice as a conscious effort. By the second interview, there was a sense that many skills were being used more regularly in practice with a greater number of communication skills mentioned as key learnings. While fewer people mentioned the theoretical methods or the communication acronyms as key learnings at T2, a number spoke of the micro-skills learned during the retreat.

Good communication skills training includes developing participants’ awareness of the communication context and skills in determining when to implement different skills to achieve an outcome appropriate for the patient and clinician [7]. Many participants in this study demonstrated a growing awareness of these issues, speaking about their emotional responses needing to be considered, awareness of their own communication styles, and being aware of when they can and cannot implement the communication skills.

Barriers to implementing the new skills were a mix of the practical considerations of time, structure of work, and to some extent expectations of colleagues. While the workplace was generally not seen as hindering good communication skills, there was recognition that some structural barriers and work routines involving other clinicians made implementation difficult. These difficulties were noted in some critical care specialties, and while they need to be confirmed, they may suggest more specific training focusing on work practices in these specialties is needed.

Several limitations need to be noted. The study did not assess patient experiences with clinicians to understand whether patients noted a difference or a benefit from healthcare professionals’ enhanced or new communication skills. Study participants were drawn from one retreat program, and findings may reflect the facilitators and participants at this retreat. More studies involving larger samples from retreat-based training programs are needed. The number of participants taking part in both interviews was relatively small. While saturation of major themes was achieved, saturation may not have been reached when assessing the key learnings from the retreat. The onset of the COVID-19 pandemic in March 2020 and the restrictions and refocus of the health system to deal with this meant clinicians’ experiences with patients at face-to-face appointments were limited by T2.

This study demonstrates that a 3-day communications skills training program utilizing a small group, learner-centric approach, and practice with simulated patients can increase health professionals’ awareness of their communication style, barriers to communicating effectively with patients, and self-efficacy for empathic communication.

### Supplementary Information

ESM 1: **Supplementary Table 1: Questions used in Interview 1 and Interview 2.** Supplementary Table 2: Exemplar quotes relating to the format of the training program. (DOCX 24 kb)

## Footnotes

1 Continuing Medical Education funding. In recognition of the need for medical staff to maintain their expertise and knowledge of current practice, medical staff at public hospitals in Victoria, Australia, receive an annual allowance to fund attendance at accredited training programs/conferences.
