# Supplementary material for: What Impact Does Participation in a Communication Skills Training Program Have on Health Professionals’ Communication Behaviors: Findings from a Qualitative Study
Source: J Cancer Educ. 2023 May 8;38(5):1600–7. doi: 10.1007/s13187-023-02305-9 (PMC10166455; doi:10.1007/s13187-023-02305-9)
Supplement: Supplementary file 1 — Supplementary Table 1: Questions used in Interview 1 and Interview 2. Supplementary Table 2: Exemplar quotes relating to the format of the training program. (DOCX 24 kb) [file 13187_2023_2305_MOESM1_ESM.docx]

Supplementary Table 1: Questions used in Interview 1 and Interview 2

| **Interview 1** | | |
| --- | --- | --- |
| Background | First what is your position and what organisation do you work for? | |
|  | Did your organisation fund you to attend the course? | |
| Organisation of Retreat | Can you tell me your overall impressions of the workshop? Probe: i) what did you think of the way the workshop was run (eg, small groups, developing scenarios, appropriateness of scenarios), ii) the facilitators ability to tailor and guide the learning of the group to help you build skills regarding communication? | |
|  | What did you think of the facilitators and the way they interacted with participants? Did you work with one more than others? | |
|  | Was there something the facilitators did in particular that helped you to learn about the different ways to communicate? What was it about their interactions or style that helped you? Do you think this was the case for all facilitators or just the one you were working with? Would others in your group have had similar experiences? | |
|  | During the retreat, simulated patients were used to demonstrate different communication scenarios and to allow participants to practice their skills. What did you think of the simulated patients? (Probe: how important for the learning? were they convincing? Was there sufficient time to practice with them?) | |
| Key learnings | What were the key learnings from the Retreat for you? | |
| Implementation | Can you tell me about how you have been able to implement these learnings into your daily clinical practice? (Probe: What are the things you find you can easily implement? What is more difficult? Why was this difficult?) | |
|  | Have you experienced any barriers from your workplace or colleagues when you have tried to implement these learnings into your daily practice? | |
|  | *For those that say they haven’t tried to implement learnings into clinical practice yet*  How do you think the training at the retreat has assisted you in your everyday clinic practice? | |
| Impact of training | How do you think your patients have responded to your new communication skills? (Probe have conversations been easier? Are they more open?) | |
|  | **For those responding positively** Could you share an example of a patient situation where the communication skills might have changed or improved your clinical practice with them? | |
|  | What do you think your organisation has gained from you undertaking the course to date? (Probe: Plans to conduct training, champion for communication skills, modelling behaviour, improved patient safety and quality of care) | |
|  | Do you think you have been able to act as a role model for effective communication skills at your organisation? Why do you say this? | |
| **Any other comments** | **Is there anything else you would like to discuss that you think is relevant to our talk today?** | |
| **Interview 2** | | |
| Key learnings | | It has been 7 or so months since you did the Retreat, what do you consider the take-home learnings from the Retreat are now? |
|  | | Are there other skills or learnings that you feel you have used in the previous 6 months? |
| Implementation | | We would like to get a sense of what people do with the skills they learn through the Retreat and how easy or difficult they find implementing these learnings in their clinical practice over the longer term.  What has been easy for you to implement consistently over the past 6 months? (Probe: Why has it been easy to adopt these learnings) |
|  | | Are there learnings you find more difficult to implement consistently? What are these? (Probe: Why do you think these are difficult to implement?) |
| Impact of training | | For those responding positively  How do you think patients/clients have responded to your new communication skills? (Probe have conversations been easier? More open? More two way?) |
|  | | Could you share an example of a patient situation where the communication skills might have changed or improved your clinical practice with them? |
|  | | How do you think your communication skills have improved the quality of care you provide to patients/clients? |
|  | | What do you think your organisation has gained from you undertaking the course to date? (Probe: Plans to conduct training, champion for communication skills, modelling behaviour, improved patient safety and quality of care) |
|  | | Have you had any opportunity to mentor or provide training regarding communication skills to other health professionals at your place of work? |
| Any other comments | | Is there anything else you would like to discuss that you think is relevant to our talk today? |
|  | | **Thank you for your help. It has been very much appreciated.** |

Supplementary Table 2: Exemplar quotes relating to the format of the training program in the areas of i) small group learning, ii) simulated patients and iii) facilitators.

| **Area** | **Exemplar Quote** |
| --- | --- |
| Small group learning | And so they were the same people for the full three days, and actually, I think that worked really well because it established a trust in the group. And it meant that we knew how each other worked and people were quicker to let down their guard and just be more authentic and give things a try because they weren’t constantly being on show to new people. (Doctor) |
|  | … I really enjoyed the fact that the majority of the work was in small groups. I really enjoyed my group. We were a really mixed bunch and very experienced clinicians. (Doctor) |
|  | I loved the small groups and how much time we spent with our facilitator and [being in the] same group all the time, we all really noticed how we were evolving, I guess over the few days. Because we were in the same group. At first I thought, it might be too much to be in the same group all the time. But no, it was excellent. (Nurse/Allied Health) |
| Simulated patients | I've never worked with a patient simulator before and it absolutely made the role play and learning feel a lot more realistic rather than another participant within the training trying to pretend to be a patient as such. So I absolutely – that’s where I got the most out of the training was the fact that the role plays were utilized with a patient simulator to make it as real as possible. I thought that was fantastic. (Nurse/Allied Health) |
|  | They [actors] were absolutely exceptional…... I thought it would be a whole lot of different scenarios and it was actually really valuable to follow the one patient through and work on different aspects of the conversations that needed to be had as part of that. (Doctor) |
|  | Really good. I think it adds a lot. I think because they were actors, they got into the role very well. And the fidelity is very high. You would almost not know. You could convince yourself you're in that room. (Doctor) |
| Facilitators | The facilitator did a fantastic job of moving things on, keeping things on track, keeping firm boundaries and not allowing side-track conversation. (Doctor) |
|  | [The facilitator] made us feel really comfortable to try different things and that it was a learning environment to just try things and see how they worked. (Doctor) |
|  | She didn’t just give us answers – she made us work through what our questions were and how we could answer our own questions. (Nurse/Allied Health) |
